# Children’s Personal Exposure Measurements to Extremely Low Frequency Magnetic Fields in Italy

**DOI:** 10.3390/ijerph13060549

**Published:** 2016-05-31

**Authors:** Ilaria Liorni, Marta Parazzini, Benjamin Struchen, Serena Fiocchi, Martin Röösli, Paolo Ravazzani

**Affiliations:** 1 CNR Consiglio Nazionale delle Ricerche, Istituto di Elettronica e di Ingegneria dell’Informazione e delle Telecomunicazioni IEIIT, Piazza Leonardo da Vinci, Milano 20133, Italy; marta.parazzini@ieiit.cnr.it (M.P.); serena.fiocchi@ieiit.cnr.it (S.F.); paolo.ravazzani@ieiit.cnr.it (P.R.); 2 Department of Epidemiology and Public Health, Swiss Tropical and Public Health Institute, Basel 4002, Switzerland; benjamin.struchen@unibas.ch (B.S.); martin.roosli@unibas.ch (M.R.); 3 University of Basel, Basel 4003, Switzerland

**Keywords:** extremely low frequency magnetic fields (ELF-MF), personal measurement, children, exposure assessment, power line, transformer

## Abstract

Extremely low frequency magnetic fields (ELF-MFs) exposure is still a topic of concern due to their possible impact on children’s health. Although epidemiological studies claimed an evidence of a possible association between ELF-MF above 0.4 μT and childhood leukemia, biological mechanisms able to support a causal relationship between ELF-MF and this disease were not found yet. To provide further knowledge about children’s ELF-MF exposure correlated to children’s daily activities, a measurement study was conducted in Milan (Italy). Eighty-six children were recruited, 52 of whom were specifically chosen with respect to the distance to power lines and built-in transformers to oversample potentially highly exposed children. Personal and bedroom measurements were performed for each child in two different seasons. The major outcomes of this study are: (1) median values over 24-h personal and bedroom measurements were <3 μT established by the Italian law as the quality target; (2) geometric mean values over 24-h bedroom measurements were mostly <0.4 μT; (3) seasonal variations did not significantly influence personal and bedroom measurements; (4) the highest average MF levels were mostly found at home during the day and outdoors; (5) no significant differences were found in the median and geometric mean values between personal and bedroom measurements, but were found in the arithmetic mean.

## 1. Introduction

Leukemia is the most common type of childhood cancer, accounting for 30% of all cancers diagnosed in children younger than 15 years old. The leukemia annual incidence rate in developed countries is approximately 50 children per one million children at risk [1], with incidence peaks at ages two to six years. Little is known about its etiology.

Apart from some rare genetic syndromes, the only other identified risk factors are exposure to ionizing radiation and high birth weight. Extremely low frequency magnetic fields (ELF-MF) have been studied as a possible risk factor since late 1970s [2]. In this period, and particularly in the last 10–15 years, several meta-analyses have been published that pooled some of the available case-control studies in which ELF-MF levels were estimated either by measurements performed in the children’s bedroom or by calculating the magnetic field, and combined various exposure indices [3,4,5]. They found a statistically significant increase of the relative risk for childhood leukemia in the groups clustered as exposed to ELF-MF levels above 0.3 μT [3] or 0.4 μT [4] compared with the groups exposed at higher levels. Also on the basis of these results, in 2002 the International Agency for Research on Cancer (IARC) concluded that ELF-MF should be classified as “possibly carcinogenic to humans” [6]. A possible increase of risk has been found by [7] for overhead powerlines. On the contrary, no association has been found related to the distance from underground cables [8].

In that perspective, the characterization of the ELF-MF exposure in children is crucial for any health risk assessment process focused on the possible association of the magnetic field (MF) exposure and childhood leukemia. This should be done not only measuring the MF amplitudes, but also focusing on the MF temporal exposure pattern, which strongly depends on the children’s behavior, habits, spaces (indoor/outdoor). This has been hypothesized to be more biological relevant than average ELF-MF exposure [9] and can be done only by personal measurements. So far, only a few studies provided data about personal exposure of children in Europe [10,11,12,13], as the majority of those studies performed in North America [14,15,16,17,18,19] and Asia [20,21,22]. Earlier studies in North America and Europe focused either on adults [23,24,25,26,27,28,29,30,31,32,33,34,35] or consisted of measurements performed only in the child’s bedroom [36,37].

The aim of this study is to estimate the levels and the temporal patterns of children’s personal exposure to ELF-MF in an urban area (Milan) in Italy.

## 2. Materials and Methods

The measurements processed in this study are the Italian part of the data collected during the personal measurement study performed in the European project FP7 ARIMMORA—Advanced Research on Interaction Mechanisms of electroMagnetic exposures with Organisms for Risk Assessments. The analysis of the whole data set is already published in [13].

### 2.1. Study Protocol

The measurements have been conducted in the Milan area from April 2012 to December 2013 in Italy, using the study protocol for personal ELF-MF measurements described in [13] on a childhood population from five to 13 years old.

In detail, 86 children in the age 5–13 years old were recruited in the town of Milan (urban area) and surrounding areas (suburban area). The study population was not a random sample of children, but children with potentially higher exposure were oversampled to achieve the following three groups: (1) “PL” (Power Line) group: children living or attending a school within 200 m of an overhead high voltage power line at 50 Hz (from 132 kV to 380 kV), or within 50 m of a high voltage underground cable (220 kV); (2) “TRANSF” (Transformer ) group: children living in a building with a built-in transformer station, or at least with the transformer attached to a wall of the building; (3) “OTHER” group: children who do not satisfy the conditions of recruitment of the first two groups.

Children have been recruited through contacting schools and personal contacts with volunteer families, living in the town of Milan and surrounding areas. All subjects gave their informed consent for inclusion before they participated in the study. The study was conducted in accordance with the Declaration of Helsinki, and the protocol was approved on 20 April 2012 by the Ethics Committee of ASL Milano (EU Project ARIMMORA, FP7-ENV-2011, Grant Agreement 282891, October 2011–March 2015).

The three groups have been harmonized in terms of age and gender in order to account for different daily habits among children of different ages and genders. Seasonal variations in exposure have been suggested by [38]. Therefore, to investigate that issue, a first personal measurement has been performed in the months from May to October (for simplicity called “summer period” in the rest of the paper) when the heating systems in the houses are turned off, while the second personal exposure measurement has been carried out in the period from November to April (“winter period”) on the identical population. In Table 1 the number of study participants is indicated, clustered per age, gender, measurement group, and urbanity. Families were asked to perform an exposure measurement during the weekdays (two full days of personal measurements plus one a bedroom measurement on one day) in a season and one measurement during the weekend (three full days of personal measurements, one of which corresponds with when the child goes to school, plus a bedroom measurement on one day) in the other season to have information of the exposure during the whole week for each child. In Table 2 the number of children divided with respect to the measurements performed over weekdays or weekends is reported for each season. The subdivision of children among weekday and weekend measurements in each season was done on the basis of families’ organizational reasons. Among all the children, eight of them performed the measurement only during the week-days, whereas another eight only performed them on the weekend in both seasons due to family matters.

Measurements were conducted with portable EMDEX II meters (broadband frequency range 40–800 Hz, harmonic frequency range 100–800 Hz, sensitivity range from 0.01 to 300 μT). This is a programmable data-acquisition meter that measures the orthogonal vector components of power-frequency magnetic fields through its internal sensors. Measurements are stored in the meter’s memory and later transferred to a PC for storage, display, and analysis. The sample rate of the device for this study has been set to 30 s. During each of the measurement periods the children carried with them an EMDEX II device (Patterson, CA, USA). for two or three full days (48–72 h), accompanied by a GPS logger (Qstarz BT-Q1000XT GPS data logger, Taipei, Taiwan). Assisted by their parents, they filled in an *ad hoc* time-activity diary, supplementing the measurements with information on location and behavior of the children. The GPS data is provided in order to verify the correctness of the information provided in the time-activity diary. Additionally, parents were asked to fill in a questionnaire to provide information about both the house that they lived in and possible exposure relevant factors. Bedroom measurements were also conducted within the personal measurement period. These measurements were done before and/or after the 48/72 h of personal measurements in order to obtain at least 24-h of measurements with the meter fixed in the bedroom.

During the personal measurements the child carried the EMDEX II device in a backpack and left it in her/his schoolbag when she/he was at school. On the contrary, at home the device remained, as much as possible, close to the child, who carried it when moving in different rooms. During the bedroom measurements the EMDEX device was placed as close as possible to the child, while avoiding putting it in close proximity to ELF-MF sources (e.g., alarm clocks), in order to reduce any possible confounding measurements.

### 2.2. Verification of the EMDEX II Devices

The correct functioning of the EMDEX II devices was checked against a calibration standard on the basis of a verification protocol developed by IEIIT-CNR after each season. The verification was performed at the laboratories of the Foundation for Research on Information Technologies in Society (IT’IS, Zurich, Switzerland) by means of a certified AMCC Helmholtz coil, which generates a well-defined and homogeneous field in its center region in the range of 0.1–77 A/m (0.12–98 µT). The device under test (DUT) (*i.e.*, each EMDEX device) was positioned with each axis aligned to the AMCC axis in the center of the coil for the measurements. The measurements were taken inside a steel shielded room with the AMCC coil mounted on a wooden table. The instruments involved were at about 1 m distance to limit the interfering magnetic field. The environmental noise level was measured with each DUT at the beginning of the verification and did not exceed a level of 0.01 µT. The field levels inside the coil were chosen considering the field levels used during the calibration by the manufacturer (*i.e.*, 0.099 µT, 1.998 µT, 19.980 µT, 96.758 µT). The fields were measured with each EMDEX II device using three different input signals (50 Hz sine, 200 Hz sine, 50 Hz sawtooth), registering each field component (x,y,z) in the broadband and harmonic frequency range.

In both the verifications, after the summer and winter periods, respectively, the EMDEX II devices showed measurement errors always within the accuracy of the device established by the manufacturer (±10%) and, therefore, no further calibration was necessary.

### 2.3. Data Management and Statistical Analysis

After the collection of all ELF-MF exposure measurements, the measurements were split into 24-h personal measurements and 24-h bedroom measurements. As previously stated, the weekday measurements consisted of two days of personal measurements, while for the weekend three days of personal measurements were collected. As per the bedroom measurements, measurements in the children’s bedrooms were either collected over a 24-h period with the measurements performed before or after the personal measurements, or were obtained by combining part-day measurements up to one full day collected in the children’s bedrooms before and after the personal measurements.

From 86 children’s exposure measurements collected in both seasons, 208 24-h personal measurements (68 from PL group, 61 from TRANSF group, and 79 from OTHER group) and 82 24-h bedroom measurements have been collected in the summer period, while 220 24-h personal measurements (69 from PL group, 61 from TRANSF group, and 90 from OTHER group) and 84 24-h bedroom measurements (26 from PL group, 25 from TRANSF group, and 33 from OTHER group) have been collected in the winter period. Measurements were excluded if they could not cover the whole 24-h period.

Median, geometric, and arithmetic mean values have been calculated for each study participant and each 24-h personal and bedroom measurements in both the broadband and harmonic frequency ranges measured by the EMDEX device. These measures have been used in order to provide a full description of the collected data, since it is not well known which of them could better describe a possible biological effect [11]. The median value permits to find the value that divides the ranked data in two halves and represents a useful descriptive measure; however, in order to provide more statistical properties, the arithmetic mean has been also calculated. Furthermore, since the arithmetic mean could be more sensitive to the presence of outliers, the geometric mean has also been taken into account.

The median values over 24-h of both personal and bedroom measurements have been compared with 3 μT, which was used as it is the minimum permitted level of exposure (quality target) at 50 Hz by Italian law [39]. In detail, this quality target is expressed as the median value over 24-h to be used for the design of new plants in proximity to children’s playgrounds, residential dwellings, school premises, and in areas where people are staying for 4 h or more per day, and also for the design of new premises and new areas close to already existing power lines in order to minimize the exposure to ELF-MF due to the risk of possible long-term effects.

Furthermore, since in [4] the level of 0.4 μT was defined with respect to the geometric mean over 24-h, in this study 24-h geometric means of both personal and bedroom measurements have separately been compared to 0.4 μT.

The effect of some factors on the ELF-MF exposure was investigated by means of linear mixed models [40], using the statistical software R [41]. In detail, the covariables “study group” (PL, TRANSF, OTHER), “season” (summer and winter), and “type of measurement” (personal and bedroom measurement) were used as explicative of possible fixed effects, while the random effects were due to the repeated measurements within one child and to children within the same family. The dependent variables were the summary measures (median, geometric mean, and arithmetic mean) calculated separately over 24-h of personal and bedroom measurements. Those dependent variables were log-transformed in order to satisfy the linear mixed model assumptions. After that, the back-transformed model coefficients were used to explain the factorial changes of ELF-MF exposure levels.

Finally, on the basis of the information provided in the daily time activity diary, each 24-h personal measurement has also been split and the exposure has been analyzed separately at home during day and night (“hd + hn”), at home during the day (“hd”), at home during the night (“hn”), at school (“s”), and outdoors (“o”) in order to account for possible variations in exposure patterns correlated with the different environments in which the child stayed during the personal measurement day. The boundaries between day and night, when the child is at home, correspond to the hours the child goes to bed and gets up as declared in the time activity diary and, therefore, they are different for each personal measurement.

## 3. Results

**Figure 1:**
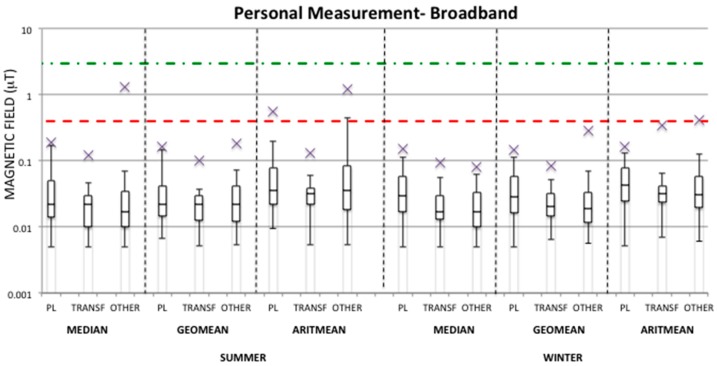
Distribution of the median, geometric mean (geomean), and arithmetic mean (aritmean) of the magnetic field (μT) in the broadband frequency range (40–800 Hz) indicated as a box-plot for personal measurements for each group (PL, TRANSF, OTHER) in both summer and winter periods. The bars represent the 25th percentile, median, and 75th percentile values, while the whiskers are the minimum and 95th percentile values. The star represents the maximum value. The red dashed line is at 0.4 μT. The green dot-dash line at 3 μT is the 24-h median value established by Italian law [36] as the quality target. The distribution values are indicated in Appendix Table A1.

**Figure 2:**
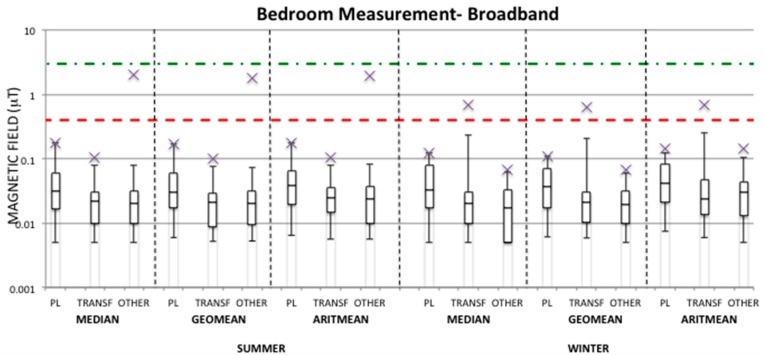
Distribution of the median, geometric mean, and arithmetic mean of the magnetic field (μT) in the broadband frequency range (40–800 Hz) indicated as a box-plot for bedroom measurements in both summer and winter periods for each group (PL, TRANSF, OTHER). The distribution values are indicated in Appendix Table A2.

Figure 1 and Figure 2 show box-plots of the calculated summary measures (median, geometric mean, and arithmetic mean) of the ELF-MF over 24-h personal measurements and 24-h bedroom measurements, respectively, for each season and each group (PL, TRANSF, OTHER) in the broadband frequency range (40–800 Hz).

From the Figure 1 and Figure 2 it can be observed that the median values over 24-h for both personal and bedroom measurements in each season and group are well below the quality target fixed at 3 μT by Italian law [36]. Indeed, the maximum values found are up to 1.31 μT and 2.1 μT in the OTHER group for personal and bedroom measurements, respectively, in the summer period, whereas during the winter period the highest maximum values are 0.15 μT for the PL group in regards to personal measurements and 0.69 μT for the TRANSF group in regards to bedroom measurements. The higher exposure observed in the OTHER group during the summer period with respect to the other groups and season is due to one case, whose high exposure was always observed at home especially during the night hours in the personal measurement days and during the whole time the meter device was fixed for bedroom measurements. However, this high exposure for the same child was not found during the winter period, and therefore a possible explanation of these high MF levels could be due to a wrong position of the EMDEX device (e.g., very close to a MF source).

Furthermore, looking at the geometric mean exposure in personal measurements, the maximum values in all groups are well below 0.4 μT fixed in [4]. In bedroom measurements (Figure 2) the OTHER group in the summer period and the TRANSF group in the winter period show maximum geometric mean values above 0.4 μT and up to 1.82 μT and 0.63 μT, respectively. As previously discussed for the median value, the maximum geometric mean value found in the OTHER group in bedroom measurement always corresponds to the same case, and also the maximum exposure level found in the TRANSF group depends to one case. Therefore, those maximum values could be considered as outliers potentially caused by a mistake in the position of the measurement device. For all groups the 95th percentiles of the distribution of geometric means lie well below 0.4 μT for both personal and bedroom measurements.

Even though the level of 0.4 μT has been defined in [4] on the basis of the geometric mean values over 24-h, a comparison can also be done with the median and arithmetic mean values. For both personal and bedroom measurements in the summer period both median and arithmetic mean values have always been found below 0.4 μT in all groups apart from the OTHER group for both metrics and the PL group for the arithmetic mean value. During the winter period the OTHER group is slightly over 0.4 μT in the arithmetic mean of personal measurements, while for the bedroom measurements the TRANSF group always presents maximum median and arithmetic mean values higher than 0.4 μT. However, in almost all cases the 95th percentile of median and arithmetic mean values are well below the level of 0.4 μT.

The highest 95th percentile value of the distribution of each summary measure for personal measurements (shown in Figure 1 as upper whiskers) has been observed in almost all cases in the PL group; this is also true in observing the 95th percentile values of summary measures in bedroom measurements during the summer period, while this result was not observed in the winter period, in which the TRANSF group is the highest exposed.

Finally, observing the condition of maximum exposure in the PL group, in almost all cases the maximum MF levels found for both personal and bedroom measurements in all seasons have been found in children living at a distance <50 m from an overhead high voltage power line. 

**Figure 3:**
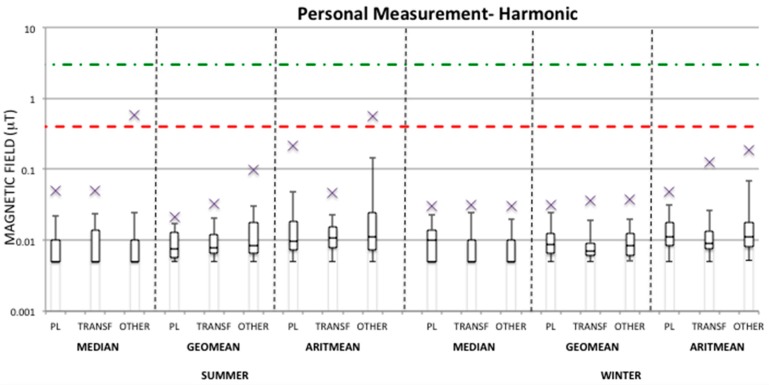
Distribution of the median, geometric mean, and arithmetic mean of magnetic field (μT) in the harmonic frequency range (100–800 Hz) indicated as box-plots for personal measurements in both summer and winter periods and in each group (PL, TRANSF, OTHER). The distribution values are indicated in Appendix Table A3.

**Figure 4:**
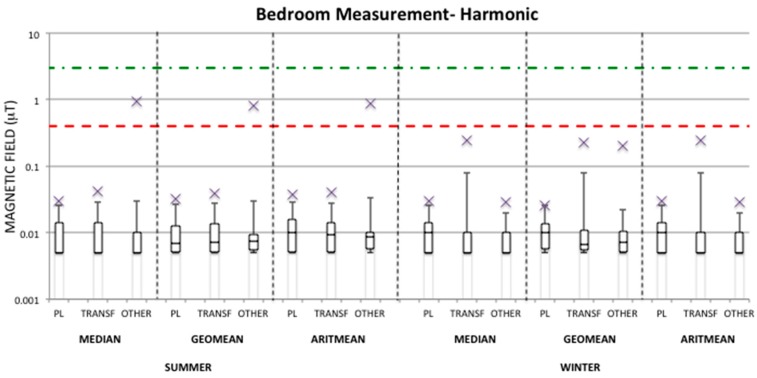
Distribution of the median, geometric mean, and arithmetic mean of magnetic field (μT) in the harmonic frequency range (100–800 Hz) indicated as box-plots for bedroom measurements in both summer and winter periods and in each group. The distribution values are indicated in Appendix Table A4.

The same analysis has also been performed in the harmonic frequency range (100–800 Hz) measured by the EMDEX device for both personal and bedroom measurements (Figure 3 and Figure 4) in each season and group. It can be observed that in this case the maximum harmonic values are always substantially lower than the ones observed in the broadband frequency range for both personal and bedroom measurements. Therefore, the median values over 24-h in all seasons and groups are well within the quality target of 3 μT of the Italian law with the maximum value found of 0.59 μT and 0.93 μT for personal and bedroom measurements, respectively, in the OTHER group during the summer period, while during the winter period the maxima found are 0.03 μT in all groups for personal measurements and 0.24 μT in the TRANSF group for bedroom measurements in the winter period. Also, the maximum geometric mean values in both personal and bedroom measurements are lower than 0.4 μT, apart from the maximum geometric mean value found within the OTHER group in bedroom measurements in summer. For this group, the median and arithmetic mean values of personal and bedroom measurements in summer are also >0.4 μT. As mentioned above this observation could be seen an outlier considering that the 95th percentile value of the geometric mean for this group is well below the fixed level of 0.4 μT.

In order to summarize the information about children’s exposure over each 24-h, in Table 3 the average values of the obtained summary measures of personal and bedroom measurements are shown for each group and in each measurement period for both broadband and harmonic frequency ranges.

**Figure 5:**
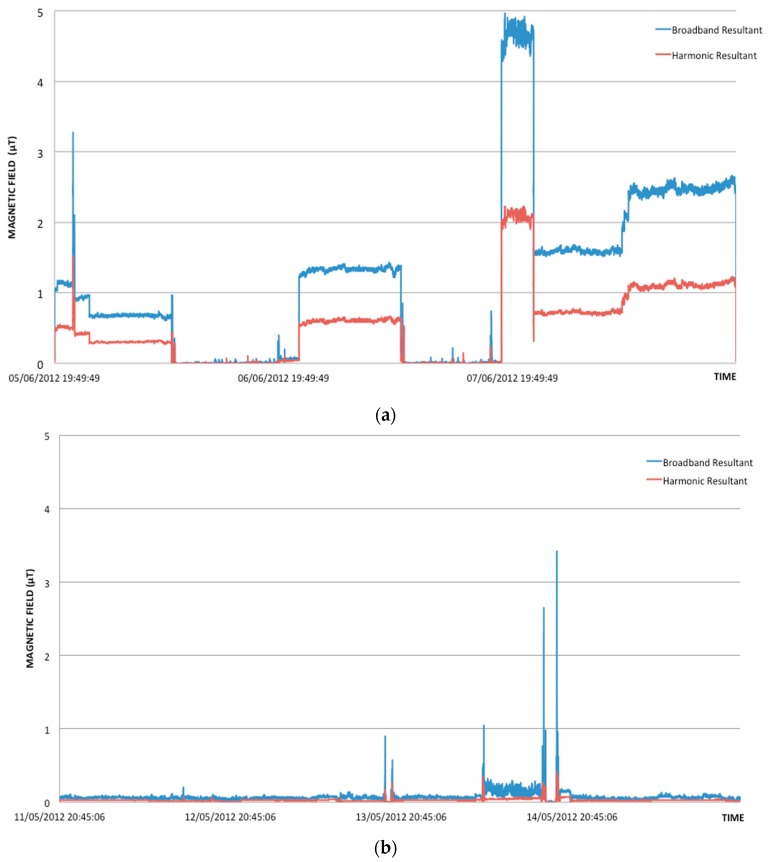
Examples of exposure measurements. (**a**) Trend of the magnetic field (μT) measurements over time performed by a child within the OTHER group during the summer period, in which high exposure has been observed; (**b**) typical trend of magnetic field measurements over time of another child always belonging to the OTHER group performed in the summer period as well.

From Table 3 it can be observed that, on average, the TRANSF group is always the lowest exposed in both seasons, unless in the bedroom measurements of the winter period. Furthermore, the OTHER group presents average summary measures higher in the summer period than in the winter period for both personal and bedroom measurements for both broadband and harmonic ranges. This is due to the fact that among the measurements collected for the OTHER group during the summer period there is a case that presents a level of exposure higher than the others in the same summary measure distribution (see Figure 1, Figure 2, Figure 3 and Figure 4), which was never observed during the winter period. Since this observation was not omitted from the analysis, it has influenced the estimation of the mean value of the summary measures for the OTHER group. For example, by removing this observation from the summer measurements of the OTHER group, one could obtain an average geometric mean of 0.031 μT and 0.023 μT for personal and bedroom measurements, respectively, in the broadband frequency range, and of 0.012 μT and 0.009 μT, respectively, in the harmonic frequency range. Figure 5 represents the one case in which the exposure levels are the highest within the OTHER group in the summer period in comparison to a typical case found within the same group and in the same season.

Finally, from Table 3 it can be observed that average values of the magnetic field summary measures over 24-h in the harmonic frequency range are substantially lower than the broadband ones. For this reason this frequency range will be neglected in the following analysis.

Linear mixed models were used to account for possible effects on the ELF-MF exposure levels due to the study group (PL, TRANSF, OTHER), the seasonal period, and the measurement type (*i.e.*, personal or bedroom).

From Table 4 it can be observed that no significant effect is introduced when ELF-MF exposure is analyzed with respect to the “study group”.

In general, children within the PL and OTHER groups are always more exposed than children belonging to TRANSF group, as previously observed above. Seasonal variations have no significant effect on exposure, as well as measurement type apart from a significant difference in the arithmetic mean of personal and bedroom measurements, resulting in the former showing an, on average, around 30% higher exposure.

**Figure 6:**
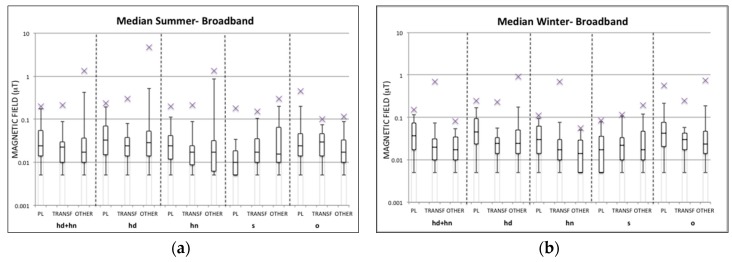

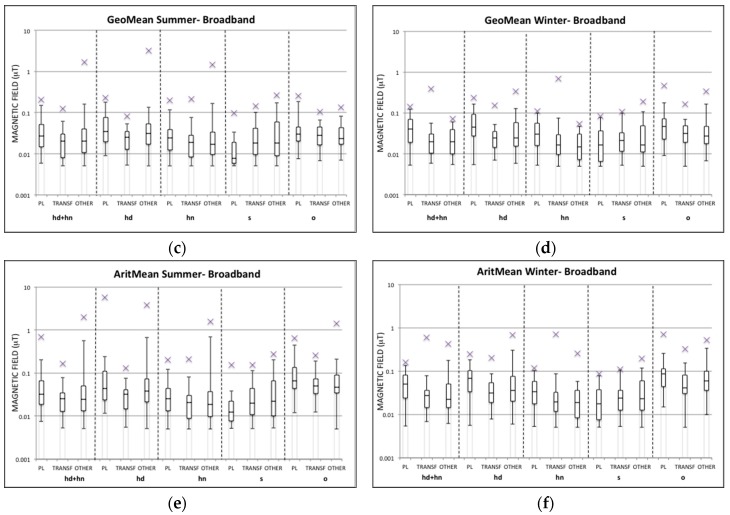
Distribution of median, geometric mean, and arithmetic mean of the magnetic field (μT) in different environments for each personal measurement day for each group and season in the broadband frequency range. (**a**) Distribution of the median of the magnetic field in different environments during the summer period; (**b**) Distribution of the median of the magnetic field in different environments during the winter period; (**c**) Distribution of the geometric mean of the magnetic field in different environments during the summer period; (**d**) Distribution of the geometric mean of the magnetic field in different environments during the winter period; (**e**) Distribution of the arithmetic mean of the magnetic field in different environments during the summer period; (**f**) Distribution of the arithmetic mean of the magnetic field in different environments during the winter period; Legend: hd + hn = at home during day and night; hd = at home during day; hn = at home during night; s = at school; o = outdoors. The distribution values are indicated in Appendix Table A5.

Once children’s measurements were analyzed over each 24-h period, an analysis of the personal measurements in each group and season was conducted with respect to the different environments in which the child stayed during each measurement day, identified through the information provided in the time-activity diary. Figure 6 presents the exposure within different activities as box-plots of the summary measures (median, geometric mean, arithmetic mean) of the magnetic field in each season and group relative to each environment (*i.e.*, at home during day and night (hd + hn), and separately (hd and hn), at school (s), and outdoors (o)) in which the child stayed during each 24-h personal measurement. The outdoor activities also include the time a child spent on public transport (buses, tube, and tram) and in the car.

On average, it was calculated that children are used to spending 42% of the day at home during the night, 28% during the day, 18% of the daily time at school, and 10% outdoors. The remaining time of about 2% was indicated by parents as time spent indoors in a place different from home; however, it was not considered in this analysis. In general, children spent more time at home in the winter period than in summer (+4%), while in the summer children spent more time outdoors than in the winter (+3%).

Figure 6 shows similar distributions for all summary measures of the magnetic field in the same environment for each group and in both seasons. For this reason the results are discussed only in terms of the trend of the geometric mean value.

In general, within each environment less than 5% of geometric means were above 0.2 μT across all groups and seasons. The highest exposed groups with 95th percentile higher than 0.1 μT across the different environments were always the PL and OTHER groups. In detail, for the PL group the highest exposure was observed when children were at home during the day and outside. Specifically, the highest maximum values found at home were in almost all cases observed in children living at a distance <50 m from overhead high voltage power lines. For the OTHER group the highest exposure in the summer period was at home and at school, and in the winter period it was at home during the day and outside.

This pattern can also be observed for the average values of each summary measure reported in Table 5 for each group and season in each environment for the broadband frequency range.

## 4. Conclusions

In this study ELF-MF personal exposure measurements have been performed in the town of Milan (Italy) on 86 children in order to make a comprehensive collection of exposure data, made of personal and bedroom measurements, in which the information about ELF-MF exposure levels have been correlated to children’s daily activities. Children were divided into three different groups (PL, TRANSF, OTHER), classified with respect to the vicinity to well-known ELF-MF sources (*i.e.*, power lines and built-in transformers), and they were measured twice in two different periods to account for possible seasonal variations due to changes in electric consumption. The study population was not a random sample, since children with potentially higher exposure were oversampled.

Several summary statistics (median, geometric mean, arithmetic mean) of the measured magnetic field have been taken into account, being so far not well known which could be the best metric to describe a possible biological effect [11].

A comparison of the median values of the magnetic field over each 24-h of personal and bedroom measurements with respect to the 24-h median quality target of 3 μT has been carried out. Those values were always found below the target limit with a maximum of 1.31 μT and 2.1 μT for personal and bedroom measurements, respectively.

Furthermore, the geometric mean values over 24-h of both personal and bedroom measurements were compared with the value of 0.4 μT, which is the level used in epidemiological studies to cluster ELF magnetic field levels investigating the possible association of exposure to childhood leukemia. Also, in this case the 24-h personal and bedroom measurements showed geometric mean values less than 0.15 μT and 0.21 μT for at least 95% of the children in both seasons and in all groups.

The harmonic frequency range (100–800 Hz) measured by the EMDEX II device resulted in measurements that were significantly lower than the measurements performed in the broadband frequency range (40–800 Hz) with maximum 24-h median values of up to 0.59 μT and 0.93 μT for personal and bedroom measurements, respectively, meaning that the main contribution to the exposure is due to 50 Hz MFs. This result is in line with the study by Fiocchi and colleagues [42].

Seasonal variations have no effect on ELF-MF exposure levels. This could be due to the fact that in Italy the power consumption is comparable in the different seasons analyzed (e.g., due to the use of the heating in the winter period and of the air conditioning in the summer period).

In addition, the type of measurements (*i.e.*, personal or bedroom measurement) has no significant effect on the median and geometric mean of ELF-MF exposure over 24-h. An effect of the type of measurement has been observed on the arithmetic mean, resulting in the personal measurements showing, on average, ELF-MF levels higher than those resulting from bedroom measurements.

Finally, although the level of exposure measured for the TRANSF group was lower compared to the PL and OTHER groups, the effect of belonging to a specific group has not been found to be statistically significant. The relative low level of exposure in the TRANSF group with respect to the values found in other studies [43,44,45] could be explained by considering that in our case the children have not been recruited due to them living in close proximity to the transformer, but only because they are living in a building in which the transformer exists. 

For the PL group, the results obtained by the analysis of the distribution of summary measures and by the linear mixed models are in line with the results already published about children’s personal measurements in Europe [10,11]. Those studies also took into account the influence of distance to overhead power lines on children’s exposure to ELF-MF and found exposure levels in some cases higher than the ones found in this study, even though in this study the highest MF levels in personal and bedroom measurements were also mostly found in the few available children living at a distance <50 m from high voltage power lines. In this study the criterion of children’s selection within the PL group was not to have only children living in close proximity to overhead power lines (<50 m), but also children at a distance up to 200 m or close to an underground cable, in order to have a maximum range of possible exposure scenarios. However, adopting this criterion of children’s selection based upon possible differences in the children’s exposure could have been diluted, since no specific sub-classification of children in the PL group with respect to different distances from power lines was performed in order to avoid losing the statistical power of the summary measures by inferring on a smaller number of samples. It should be noted that this study was designed to oversample potentially highly exposed subjects by including a broad definition of such a scenario, to also look at temporal pattern of people, who on average were not highly exposed, but still moderately close to a source, which they may pass from time to time. As confirmed by the results, this approach is not suitable to investigate exposure contrasts between the groups. To that latter purpose, the experimental design should be modified, using shorter distances to power lines and transformers. In future studies a larger sample of children should be measured and divided into sub-groups in order to take into account several distances to different types of overhead power lines.

Furthermore, since children belonging to the PL group were mostly found in urban areas (see Table 1), in which there is a prevalence of underground cables, whose profile of the magnetic field is known to decrease more rapidly with distance than magnetic fields from overhead power lines [46], an additional sub-group could be introduced to separately analyze the exposure of children living close to underground cables from the ones living close to overhead power lines in order to highlight other possible differences in exposure. As already suggested by Bunch and colleagues [8], this kind of analysis could also be an interesting way of separating magnetic field effects from other (non-EMF) factors only associated with overhead power lines (e.g., the production of corona ions) which could be responsible of a possible increased risk of childhood leukemia.

From the analysis of ELF-MF exposure levels in different environments in which children stayed during the personal measurement days, it was observed that the PL and OTHER group were in almost all environments the highest exposed groups, and that the highest average exposure levels were mostly found when the children were at home during the day and outside. The fact that exposure levels found for the OTHER group are comparable in some cases to the ones of the PL group represents an interesting result, which highlights that children who do not live directly in close proximity to known ELF-MF sources that are responsible of potential high exposure could also be exposed to unknown MF sources during their daily life anyway, depending on their daily activities and movements.

In conclusion, this is the first study which aimed to assess ELF-MF exposure of a sample of healthy children in Italy. The exposure levels in the town of Milan were always significantly below the quality target established by Italian law and were also found to be far lower than 0.3–0.4 μT. The study also provided useful information about exposure in several environments in which children are used to spending their time, thereby pointing out the fact that living in proximity to ELF-MF sources contributes to children’s exposure but that children’s habits and behaviors also affect their exposure as well.

## Figures and Tables

**Table 1:** Number of study participants for each measurements group (PL (Power Line) group, TRANSF (Transformer) group, OTHER group) divided per gender, age, and urbanity.

Group	Gender		Age				Urbanity	
	Male	Female	5,6	7,8	9,10	11,13	Urban	Suburban
PL	14	13	4	6	9	8	20	7
TRANSF	10	15	5	4	9	7	24	1
OTHER	19	15	8	8	12	6	30	4
Total	43	43	17	18	30	21	74	12

**Table 2:** Number of measurements performed over weekdays and weekends in each season.

Season	Week-Day	Week-End
Summer	50	36
Winter	36	50

**Table 3:** Average value of the median, geometric mean, and arithmetic mean of magnetic field in each group for personal and bedroom measurements in each season for both broadband and harmonic frequency ranges.

Measurement Type and Group	Average Value (μT)					
	Broadband			Harmonic		
Summer	Median	GeoMean	AritMean	Median	GeoMean	AritMean
**Personal**						
**PL**	0.039	0.035	0.067	0.009	0.009	0.020
**TRANSF**	0.023	0.022	0.033	0.010	0.010	0.013
**OTHER**	0.040	0.031	0.093	0.017	0.013	0.032
**Bedroom**						
**PL**	0.053	0.052	0.058	0.009	0.010	0.012
**TRANSF**	0.025	0.025	0.028	0.011	0.010	0.012
**Other**	0.086	0.079	0.086	0.038	0.034	0.037
**Winter**						
**Personal**						
**PL**	0.042	0.041	0.054	0.010	0.011	0.014
**TRANSF**	0.023	0.024	0.041	0.009	0.009	0.014
**OTHER**	0.023	0.028	0.049	0.009	0.010	0.018
**Bedroom**						
**PL**	0.048	0.045	0.053	0.012	0.011	0.012
**TRANSF**	0.049	0.047	0.058	0.018	0.018	0.018
**OTHER**	0.022	0.023	0.036	0.008	0.014	0.008

**Table 4:** Model coefficients of the chosen variables (study group, season, and measurement type) for the target variables (median, geometric mean, and arithmetic mean).

Covariables	Dependent Variables: Exposure Metrics		
	Median	GeoMean	AritMean
Group: TRANSF	0.75 (0.46; 1.22)	0.76 (0.50; 1.16)	0.74 (0.48;1.12)
Group: PL	0.97 (0.67; 1.39)	0.95 (0.69; 1.31)	0.99 (0.70; 1.40)
Season: Winter	1.00 (0.92; 1.08)	1.02 (0.95; 1.09)	0.99 (0.90; 1.08)
Measurement Type: Personal	0.95 (0.87; 1.03)	1.01 (0.93; 1.09)	1.29 (1.17; 1.43) ***

The 95% percentiles are given in brackets. The stars indicate the significance range (0.05 ≥ *p* >0.01; 0.01 ≥ *p* > 0.001; *** *p* ≤ 0.001). Reference for “study group”: OTHER; reference for “season”: summer; reference for “measurement type”: bedroom.

**Table 5:** Average value of the median, geometric mean, and arithmetic mean of personal measurements classified with respect to different environments for each group and season in the broadband frequency range.

Environment and Group	Average Value (μT)- Broadband					
	Summer			Winter		
	Median	GeoMean	AritMean	Median	GeoMean	AritMean
**hd + hn**						
**PL**	0.044	0.044	0.064	0.046	0.048	0.055
**TRANSF**	0.027	0.024	0.029	0.034	0.029	0.040
**OTHER**	0.074	0.057	0.110	0.022	0.024	0.047
**hd**						
**PL**	0.056	0.057	0.159	0.062	0.064	0.075
**TRANSF**	0.031	0.026	0.033	0.029	0.028	0.039
**OTHER**	0.123	0.082	0.154	0.064	0.044	0.080
**hn**						
**PL**	0.037	0.038	0.040	0.039	0.040	0.042
**TRANSF**	0.025	0.024	0.027	0.033	0.033	0.038
**OTHER**	0.094	0.057	0.094	0.019	0.019	0.028
**s**						
**PL**	0.016	0.015	0.018	0.024	0.024	0.026
**TRANSF**	0.031	0.031	0.035	0.032	0.031	0.033
**OTHER**	0.049	0.044	0.052	0.036	0.035	0.040
**o**						
**PL**	0.047	0.046	0.123	0.063	0.062	0.100
**TRANSF**	0.032	0.033	0.066	0.034	0.036	0.065
**OTHER**	0.027	0.032	0.084	0.053	0.045	0.096

## Appendix

**Table A1:** Distribution values of the median, geometric mean (geomean) and arithmetic mean (aritmean) of magnetic field (μT) in the broadband (Brd) frequency range (40–800 Hz) for personal measurements for each group (PL, TRANSF, OTHER) in both summer and winter periods.

Magnetic Field (μT)												
Personal_Brd	Summer Period						Winter Period					
	min	p25	p50	p75	p95	max	min	p25	p50	p75	p95	max
**Median**												
PL	0.005	0.014	0.022	0.051	0.173	0.189	0.005	0.017	0.030	0.058	0.112	0.154
TRANSF	0.005	0.010	0.022	0.030	0.046	0.121	0.005	0.013	0.017	0.030	0.057	0.093
OTHER	0.005	0.010	0.017	0.035	0.070	1.307	0.005	0.010	0.017	0.033	0.062	0.081
**GeoMean**												
PL	0.007	0.015	0.022	0.042	0.146	0.168	0.005	0.017	0.029	0.057	0.112	0.147
TRANSF	0.005	0.013	0.022	0.029	0.037	0.100	0.006	0.015	0.021	0.032	0.051	0.085
OTHER	0.005	0.012	0.022	0.042	0.074	0.182	0.006	0.012	0.019	0.033	0.069	0.285
**AritMean**												
PL	0.010	0.022	0.036	0.079	0.201	0.565	0.005	0.025	0.044	0.078	0.130	0.162
TRANSF	0.005	0.022	0.032	0.039	0.061	0.133	0.007	0.024	0.032	0.042	0.066	0.352
OTHER	0.005	0.019	0.036	0.083	0.447	1.246	0.006	0.020	0.031	0.058	0.126	0.416

Legend: p25 = 25th percentile; p50 = 50th percentile; p75 = 75th percentile. These values are represented in Figure 1 of the paper.

**Table A2:** Distribution values of the median, geometric mean (geomean) and arithmetic mean (aritmean) of magnetic field (μT) in the broadband (Brd) frequency range (40–800 Hz) for bedroom measurements for each group (PL, TRANSF, OTHER) in both summer and winter periods.

Magnetic Field (μT)												
Bedroom_Brd	Summer Period						Winter Period					
	min	p25	p50	p75	p95	max	min	p25	p50	p75	p95	max
**Median**												
PL	0.005	0.017	0.032	0.061	0.175	0.179	0.005	0.017	0.033	0.081	0.122	0.123
TRANSF	0.005	0.010	0.022	0.030	0.079	0.105	0.005	0.010	0.020	0.030	0.231	0.693
OTHER	0.005	0.010	0.020	0.032	0.078	2.060	0.005	0.005	0.017	0.033	0.063	0.066
**GeoMean**												
PL	0.006	0.017	0.030	0.059	0.168	0.168	0.006	0.017	0.036	0.069	0.109	0.109
TRANSF	0.005	0.009	0.021	0.029	0.077	0.102	0.006	0.011	0.021	0.030	0.211	0.627
OTHER	0.005	0.010	0.020	0.032	0.073	1.823	0.005	0.010	0.020	0.032	0.061	0.068
**AritMean**												
PL	0.007	0.020	0.039	0.065	0.177	0.177	0.007	0.021	0.041	0.082	0.124	0.147
TRANSF	0.006	0.015	0.025	0.035	0.078	0.103	0.006	0.014	0.024	0.047	0.250	0.678
OTHER	0.006	0.010	0.024	0.037	0.081	1.930	0.005	0.013	0.030	0.044	0.105	0.143

These values are represented in Figure 2 of the paper.

**Table A3:** Distribution values of the median, geometric mean (geomean) and arithmetic mean (aritmean) of magnetic field (μT) in the harmonic (Hrm) frequency range (100–800 Hz) for personal measurements for each group (PL, TRANSF, OTHER) in both summer and winter periods.

Magnetic Field (μT)												
Personal_Hrm	Summer Period						Winter Period					
	min	p25	p50	p75	p95	max	min	p25	p50	p75	p95	max
**Median**												
PL	0.005	0.005	0.005	0.010	0.022	0.050	0.005	0.005	0.010	0.014	0.023	0.030
TRANSF	0.005	0.005	0.005	0.014	0.023	0.050	0.005	0.005	0.005	0.010	0.025	0.032
OTHER	0.005	0.005	0.005	0.010	0.025	0.586	0.005	0.005	0.005	0.010	0.020	0.030
**GeoMean**												
PL	0.005	0.006	0.008	0.013	0.017	0.021	0.005	0.007	0.009	0.012	0.025	0.031
TRANSF	0.005	0.007	0.008	0.012	0.020	0.033	0.005	0.006	0.007	0.009	0.019	0.036
OTHER	0.005	0.007	0.008	0.018	0.031	0.099	0.005	0.006	0.008	0.013	0.020	0.038
**AritmMean**												
PL	0.005	0.007	0.010	0.019	0.048	0.211	0.005	0.009	0.011	0.018	0.032	0.049
TRANSF	0.005	0.008	0.011	0.015	0.023	0.047	0.005	0.008	0.009	0.014	0.026	0.127
OTHER	0.005	0.007	0.011	0.024	0.143	0.561	0.005	0.008	0.011	0.018	0.068	0.184

These values are represented in Figure 3 of the paper.

**Table A4:** Distribution values of the median, geometric mean (geomean) and arithmetic mean (aritmean) of magnetic field (μT) in the harmonic (Hrm) frequency range (100–800 Hz) for bedroom measurements for each group (PL, TRANSF, OTHER) in both summer and winter periods.

Magnetic Field (μT)												
Bedroom_Hrm	Summer Period						Winter Period					
	min	p25	p50	p75	p95	max	min	p25	p50	p75	p95	max
**Median**												
PL	0.005	0.005	0.005	0.014	0.026	0.030	0.005	0.005	0.010	0.014	0.026	0.030
TRANSF	0.005	0.005	0.005	0.014	0.029	0.041	0.005	0.005	0.005	0.010	0.080	0.248
OTHER	0.005	0.005	0.005	0.010	0.030	0.934	0.005	0.005	0.005	0.010	0.020	0.028
**GeoMean**												
PL	0.005	0.005	0.007	0.013	0.027	0.033	0.005	0.006	0.010	0.014	0.026	0.026
TRANSF	0.005	0.005	0.007	0.014	0.028	0.040	0.005	0.006	0.007	0.011	0.080	0.228
OTHER	0.005	0.006	0.007	0.010	0.030	0.820	0.005	0.005	0.007	0.011	0.022	0.203
**AritmMean**												
PL	0.005	0.005	0.010	0.016	0.028	0.038	0.005	0.005	0.010	0.014	0.026	0.030
TRANSF	0.005	0.005	0.009	0.014	0.028	0.040	0.005	0.005	0.005	0.010	0.080	0.248
OTHER	0.005	0.006	0.009	0.010	0.034	0.868	0.005	0.005	0.005	0.010	0.020	0.028

These values are represented in Figure 4 of the paper.

**Table A5:** Distribution values of median, geometric mean and arithmetic mean of magnetic field (μT) in different environments for each personal measurement day for each group and season in the broadband frequency range.

Magnetic Field (μT)												
Summary Measure	Summer Period						Winter Period					
**Median**	**min**	**p25**	**p50**	**p75**	**p95**	**max**	**min**	**p25**	**p50**	**p75**	**p95**	**max**
**hd + hn**												
PL	0.005	0.014	0.025	0.055	0.176	0.201	0.005	0.017	0.037	0.073	0.116	0.153
TRANSF	0.005	0.010	0.022	0.030	0.089	0.214	0.005	0.010	0.020	0.032	0.074	0.693
OTHER	0.005	0.010	0.017	0.036	0.426	1.348	0.005	0.010	0.017	0.034	0.055	0.081
**hd**												
PL	0.005	0.015	0.033	0.070	0.190	0.239	0.005	0.024	0.046	0.093	0.168	0.244
TRANSF	0.005	0.014	0.025	0.037	0.079	0.300	0.005	0.014	0.025	0.033	0.056	0.232
OTHER	0.005	0.014	0.028	0.053	0.514	4.643	0.005	0.014	0.025	0.051	0.177	0.915
**hn**												
PL	0.005	0.012	0.025	0.041	0.113	0.197	0.005	0.014	0.030	0.061	0.094	0.110
TRANSF	0.005	0.009	0.017	0.025	0.088	0.214	0.005	0.010	0.017	0.030	0.075	0.700
OTHER	0.005	0.006	0.017	0.032	0.851	1.348	0.005	0.005	0.014	0.029	0.050	0.057
**s**												
PL	0.005	0.005	0.010	0.019	0.034	0.178	0.005	0.005	0.017	0.036	0.079	0.084
TRANSF	0.005	0.010	0.017	0.035	0.105	0.152	0.005	0.010	0.022	0.033	0.109	0.114
OTHER	0.005	0.010	0.016	0.065	0.200	0.302	0.005	0.010	0.017	0.047	0.119	0.193
**o**												
PL	0.005	0.014	0.025	0.046	0.198	0.444	0.005	0.021	0.042	0.077	0.213	0.573
TRANSF	0.005	0.014	0.030	0.046	0.075	0.103	0.005	0.017	0.030	0.042	0.058	0.246
OTHER	0.005	0.010	0.017	0.033	0.087	0.115	0.005	0.014	0.023	0.047	0.184	0.750
**GeoMean**	**min**	**p25**	**p50**	**p75**	**p95**	**max**	**min**	**p25**	**p50**	**p75**	**p95**	**max**
**hd + hn**												
PL	0.006	0.015	0.027	0.052	0.151	0.211	0.005	0.019	0.041	0.070	0.124	0.141
TRANSF	0.005	0.008	0.020	0.030	0.063	0.125	0.006	0.011	0.020	0.030	0.056	0.391
OTHER	0.005	0.011	0.020	0.040	0.162	1.716	0.006	0.010	0.020	0.039	0.060	0.073
**hd**												
PL	0.009	0.019	0.035	0.076	0.180	0.229	0.005	0.028	0.045	0.094	0.164	0.239
TRANSF	0.005	0.013	0.025	0.035	0.054	0.083	0.007	0.015	0.024	0.036	0.053	0.152
OTHER	0.005	0.017	0.031	0.053	0.137	3.187	0.006	0.016	0.025	0.058	0.130	0.343
**hn**												
PL	0.005	0.012	0.024	0.038	0.118	0.200	0.005	0.016	0.031	0.056	0.100	0.110
TRANSF	0.005	0.008	0.019	0.028	0.077	0.212	0.005	0.010	0.017	0.030	0.075	0.695
OTHER	0.005	0.010	0.017	0.034	0.167	1.470	0.005	0.007	0.015	0.030	0.048	0.055
**s**												
PL	0.005	0.006	0.008	0.019	0.034	0.098	0.005	0.007	0.017	0.036	0.078	0.084
TRANSF	0.005	0.010	0.018	0.041	0.100	0.143	0.005	0.012	0.021	0.033	0.100	0.106
OTHER	0.005	0.009	0.018	0.060	0.173	0.264	0.005	0.011	0.017	0.048	0.108	0.191
**o**												
PL	0.008	0.021	0.030	0.044	0.188	0.255	0.009	0.023	0.047	0.073	0.174	0.465
TRANSF	0.007	0.016	0.028	0.045	0.066	0.104	0.005	0.020	0.032	0.049	0.071	0.164
OTHER	0.007	0.017	0.024	0.044	0.081	0.136	0.007	0.018	0.027	0.048	0.163	0.340
**AritMean**	**min**	**p25**	**p50**	**p75**	**p95**	**max**	**min**	**p25**	**p50**	**p75**	**p95**	**max**
**hd + hn**												
PL	0.008	0.019	0.032	0.067	0.204	0.683	0.005	0.024	0.050	0.081	0.137	0.157
TRANSF	0.005	0.013	0.026	0.034	0.079	0.166	0.007	0.014	0.027	0.036	0.077	0.594
OTHER	0.005	0.013	0.025	0.050	0.571	1.996	0.006	0.014	0.023	0.050	0.173	0.418
**hd**												
PL	0.012	0.023	0.043	0.112	0.237	5.751	0.006	0.034	0.068	0.103	0.181	0.249
TRANSF	0.006	0.015	0.032	0.041	0.076	0.132	0.008	0.019	0.032	0.054	0.087	0.204
OTHER	0.005	0.021	0.039	0.073	0.664	3.774	0.006	0.021	0.036	0.075	0.306	0.685
**hn**												
PL	0.005	0.013	0.026	0.043	0.122	0.201	0.005	0.018	0.033	0.059	0.103	0.116
TRANSF	0.005	0.009	0.021	0.030	0.082	0.213	0.005	0.012	0.020	0.032	0.087	0.696
OTHER	0.005	0.010	0.019	0.036	0.692	1.551	0.005	0.008	0.019	0.036	0.058	0.258
**s**												
PL	0.005	0.008	0.012	0.022	0.038	0.153	0.005	0.008	0.018	0.038	0.079	0.088
TRANSF	0.005	0.011	0.020	0.044	0.112	0.153	0.005	0.013	0.024	0.036	0.107	0.109
OTHER	0.005	0.010	0.022	0.066	0.203	0.274	0.005	0.013	0.023	0.060	0.116	0.197
**o**												
PL	0.012	0.044	0.065	0.133	0.448	0.642	0.015	0.044	0.087	0.114	0.256	0.714
TRANSF	0.012	0.033	0.050	0.074	0.190	0.260	0.005	0.030	0.042	0.082	0.153	0.321
OTHER	0.005	0.035	0.047	0.090	0.207	1.396	0.010	0.036	0.059	0.098	0.330	0.514

Legend: hd + hn = at home during day and night; hd = at home during day; hn = at home during night; s = at school; o = outdoor. These values are represented in Figure 6 of the paper.
